# Validation of the Generalized Anxiety Disorder-7 (GAD-7) and GAD-2 in patients with migraine

**DOI:** 10.1186/s10194-015-0583-8

**Published:** 2015-11-23

**Authors:** Jong-Geun Seo, Sung-Pa Park

**Affiliations:** Department of Neurology, School of Medicine, Kyungpook National University, 680 Gukchaebosang-ro, Daegu, 700-842 Jung-gu Republic of Korea

**Keywords:** Anxiety, Migraine, GAD-7, GAD-2, Validity

## Abstract

**Background:**

Psychiatric problems have been commonly reported in patients with migraine. This study investigated the reliability and validity of the Generalized Anxiety Disorder-7 (GAD-7) and Generalized Anxiety Disorder-2 (GAD-2) in patients with migraine.

**Methods:**

Subjects were recruited from a headache clinic and a neuropsychologist examined their GAD using the Mini International Neuropsychiatric Interview-Plus Version 5.0.0 (MINI). Subjects completed several instruments, including the GAD-7, the Beck Anxiety Inventory (BAI), the Migraine Disability Assessment Scale (MIDAS), the Headache Impact Test-6 (HIT-6), and the Migraine-Specific Quality of Life (MSQoL).

**Results:**

Among 146 participants, 32 patients (21.9 %) had GAD as determined by the MINI. Cronbach’s *α* for the GAD-7 and GAD-2 were 0.915 and 0.820, respectively. At a cutoff score of 5, the GAD-7 had a sensitivity of 78.1 %, a specificity of 74.6 %, a positive predictive value (PPV) of 46.3 %, and a negative predictive value (NPV) of 92.4 %. At a cutoff score of 1, the GAD-2 had a sensitivity of 84.4 %, a specificity of 72.8 %, a PPV of 46.6 %, and a NPV of 94.3 %. The scores of the GAD-7 and GAD-2 well correlated with the BAI score, the MIDAS score, the HIT-6 score, and the MSQoL score.

**Conclusions:**

The GAD-7 and GAD-2 are both reliable and valid screening instruments for GAD in patients with migraine.

## Background

Migraine is a common and often disabling neurological disorder. In a systemic review of population-based studies, the overall prevalence of migraine worldwide was 11 %, with prevalence rates of 6 % in males and 14 % in females [1]. Migraine represents a public health problem with an enormous burden to both individual patients and society [2].

Psychiatric problems have been commonly reported in patients with migraine. In a Korean hospital-based study, 36.3 % of patients with migraine had depression and 23.1 % revealed anxiety by self-report questionnaires [3]. In an Italian multicenter study, 23.1 % of patients with migraine exhibited major depressive disorder (MDD) and 18.4 % exhibited generalized anxiety disorder (GAD) as classified by a structured interview and the Mini International Neuropsychiatry Interview (MINI) [4].

Psychiatric comorbidity complicates the management of patients with headache, and the prognosis for headache treatment is poor [5]. Comorbid psychiatric disorders in patients with migraine affect the frequency and intensity of migraine attacks [6, 7]. In patients with episodic migraine, the presence of psychiatric disorders, such as anxiety or depression or both, facilitate the evolution of the headache into the chronic form [8]. Patients with migraine, anxiety, and chronic depression also had poor health-related quality of life (QOL) [9]. Among psychiatric disorders, anxiety is a common psychiatric comorbidity in patients with migraine [10–13]. Anxiety, more than depression, predicts long-term migraine persistence, headache-related disability and reduces perceptions of efficacy with acute treatment [14]. Therefore, the early diagnosis and treatment of anxiety is important for the proper management of patients with migraine. For these purposes, a simple, rapid screening instrument to detect anxiety is a prerequisite, especially in a busy clinical setting.

The Generalized Anxiety Disorder-7 (GAD-7) was developed in the USA as a valuable screening tool for detecting GAD in primary care patients [15]. The Generalized Anxiety Disorder-2 (GAD-2) is a short version of the tool that is composed of the first two questions of the GAD-7 [16]. Both the GAD-7 and GAD-2 have been widely used by general practitioners [16]. Although the GAD-7 and GAD-2 were validated in primary care patients, their usefulness in patients with migraine is unknown. Recently, the Patient Health Questionnaire-9 (PHQ-9) was validated for detecting a MDD in patients with migraine [17]. However, the cutoff score of the PHQ-9 was different from previous studies which were conducted in primary care patients. Likewise the PHQ-9, it is needed to validate the GAD-7 and GAD-2 in patients with migraine. Therefore, this study investigated the reliability and validity of the GAD-7 and GAD-2 as screening tools in patients with migraine.

## Methods

### Subjects

Subjects in this study were new patients with migraine who had consecutively visited a outpatients headache clinic in the Department of Neurology at Kyungpook National University Hospital from December 2014 to May 2015. Patients were adolescents and adults (aged 16–65 years old) who were newly diagnosed at our clinic or were already diagnosed but had not taken triptans, preventive medicines, or other neuropsychiatric agents within the last month. A diagnosis of migraine was based on the International Classification of Headache Disorders, 3^rd^ edition, beta version [18]. Patients were excluded if they were unable to cooperate in the psychiatric interview or had difficulty understanding the questionnaire because of illiteracy, mental retardation, serious medical, neurological, or psychiatric disorders, and alcohol or drug abuse. Patients with a probable migraine and those declining the interview were also excluded.

### Study design

A cross-sectional study was conducted as part of a hospital-based study that examined the impact of psychiatric disorders on migraine and migraine-associated medications, such as triptans and preventive medicines. The Institutional Review Board of Kyungpook National University Hospital approved the study. All participants gave written informed consent. Subject’s medical charts were reviewed to collect demographic, social, and clinical information for a computerized database. Sociodemographic data included age, gender, education, employment, household income (earning at least three million KRW per month, equivalent to 2800 USD per month or not), and marital status (married or unmarried, divorced, and bereaved). Clinical data included the type of migraine, age at onset, disease duration, attack frequency, attack duration, family history, and accompanying symptoms (presence of photophobia, phonophobia, or osmophobia). A family history of migraine was defined as an existing diagnosis of migraine in a lineal ascendant and/or siblings. Photophobia, phonophobia, and osmophobia were defined as hypersensitivity to light, sound, and certain odors during migraine attacks that could cause avoidance of those stimulations or aggravation of migraine symptoms. Patients were asked whether they experienced symptoms during the preceding year.

To measure the reliability of the GAD-7 and GAD-2 in eligible subjects, one neuropsychologist examined their GAD using the Mini International Neuropsychiatric Interview-Plus Version 5.0.0 (MINI) [19]. Subsequently, patients provided several self-reported questionnaires, including the Beck Anxiety Inventory (BAI) [20], the Korean version of the Migraine Disability Assessment Scale (MIDAS) [21], the Headache Impact Test-6 (HIT-6) [22], and the Migraine-Specific Quality of Life (MSQoL) [23], to examine the validity of the GAD-7 and GAD-2.

### Interview and questionnaires

#### Mini International Neuropsychiatric Interview-Plus Version 5.0.0 (MINI)

The MINI-Plus 5.0.0 is an internationally validated brief structured interview that is used extensively as a diagnostic tool for psychiatric disorders from the Diagnostic and Statistical Manual of Mental Disorders, Fourth Edition and the International Classification of Diseases-10. The reliability and validity of this instrument is well established [24], and the Korean translation is also validated [19].

### Generalized Anxiety Disorder-7 (GAD-7) and Generalized Anxiety Disorder-2 (GAD-2)

The GAD-7 and GAD-2 were designed for use in primary care patients [15, 16]. The GAD-7 consists of a self-report questionnaire that allows for the rapid detection of GAD [15]. Subjects are asked if they were bothered by anxiety related problems over the past two weeks by answering seven items on a 4-point scale. The total scores ranged from 0 to 21. At a cutoff score of 9, the GAD-7 had a sensitivity of 89 % and a specificity of 82 % for detecting GAD compared with a structured psychiatric interview [15]. The GAD-2 is a short version of the tool that is composed of the first two questions of the GAD-7 [16]. At a cutoff score of 2, the GAD-2 had a sensitivity of 86 % and a specificity of 83 % for detecting GAD [16]. The GAD-7 was translated into the Korean language, and was freely downloadable on the Patient Health Questionnaire website (www.phqscreeners.com) [25]. The translated version was translated back into English by a Korean English teacher. Finally, the two versions were compared by a native English speaker who concluded that they were identical. Thereafter, we administered it to 20 Korean PWE for the evaluation of potential problems in comprehension or cultural differences. No further adaptations were required.

### Beck Anxiety Inventory (BAI)

The BAI is a 21-item self-report measure of anxiety severity. The scale consists of 21 items, each describing a common symptom of anxiety. The respondent is asked to rate how much he or she has been bothered by each symptom over the past week on a 4-point scale from 0 to 3. The following cutoff points were used: 0–21, normal; 22–26, mild disturbance; 27–31, moderate disturbance; and 32–63, severe disturbance. The Korean version of the BAI has been validated [20]. Those who scored more than 21 points on the BAI were considered to have anxiety symptoms. Cronbach’s α was 0.9.

### Migraine Disability Assessment Scale (MIDAS)

The Korean version of the MIDAS, a 5-item questionnaire that was designed to evaluate disability during the previous three months, was used in this study [21]. Patients were asked to report decreased performance in the domains of work/school, household work, and family/social activities. Scores (0–27) measure the overall level of disability: Grade I (0–5), Grade II (6–10), Grade III (11–20), and Grade IV (above 21). Cronbach’s α was 0.75.

### Headache Impact Test-6 (HIT-6)

The HIT-6 was developed in the United States to measure a wider spectrum of headache-induced burden [26]. Items in the HIT-6 cover several domains: pain, social functioning, role functioning, vitality, cognitive functioning, and psychological distress. Each item is answered on a 5-point Likert scale (6 = never, 8 = rarely, 10 = sometimes, 11 = very often, 13 = always). The total scores ranged from 36 to 78; larger scores indicate a greater impact. For interpretation, HIT-6 scores are categorized in four groups: scores ≤49 indicate little or no impact, scores between 50 and 55 indicate some impact, scores between 56 and 59 indicate a substantial impact, and scores ≥60 indicate a severe impact [27]. The Korean version of the HIT-6 was validated and Cronbach’s α was 0.85 [22].

### Migraine-Specific Quality of Life (MSQoL)

The MSQoL developed by Wagner et al. and is a valid and reliable tool for clinical migraine research [28]. A Korean translation of this 25-item questionnaire has been validated [23]. The items are rated on a 4-point scale (1–4). The total scores ranged from 25 to 100. A lower total score indicates a poorer QOL. Cronbach’s α was 0.93.

### Statistical analyses

The Statistical Package for the Social Sciences (SPSS version 21.0) was used for data analysis. The Med Calc 8.0 was used to perform receiver operating characteristic (ROC) analyses to measure sensitivity, specificity, positive predictive values (PPVs) and negative predictive values (NPVs) for a range of cutoff scores of the GAD-7 and GAD2 with respect to the diagnoses of GAD by the MINI-Plus 5.0.0. Optimal cutoff scores were also computed using criteria that minimize the Euclidean distance from point (sensitivity and specificity) to point in the x-y plane. The descriptive statistics are presented as counts, percentages, means, and standard deviations. Independent t-tests, Mann-Whitney U tests, and Chi-square tests were used to compare continuous or categorical variables. Cronbach’s α was computed to ascertain internal consistency and was recalculated after items were removed. Nonparametric correlations (Spearman’s *ρ*) were used to determine the validity of the GAD-7 and GAD-2. The level of statistical significance was set at *p* < 0.05.

## Results

Of the 207 patients who consecutively visited a headache clinic, 61 were excluded because of probable migraine (*n* = 23), taking preventive medicine for migraine or psychotropic agents (*n* = 10), illiteracy (*n* = 6), age older than 70 (*n* = 4), and refusal to take part in the study (*n* = 18). The 146 remaining patients were eligible for this study. According to the MINI, 32 patients (21.9 %) were diagnosed with GAD. The relationships between GAD and demographic, clinical, and psychosocial characteristics are listed in Table 1. There were no significant differences in demographic characteristics. Among clinical characteristics, patients with GAD were more likely to have a phonophobia; this likelihood was statistically significant. Patients with GAD exhibited significantly higher scores on the GAD-7, the BAI, and the HIT-6, a lower score on the MSQoL than those without GAD.

**Table 1:** Demographic, clinical, and psychosocial characteristics of the eligible subjects with respect to current GAD as determined by the MINI-Plus 5.0.0

	Mean ± SD (range) or number (%)		
	No GAD	GAD	
Characteristics	(*n* = 114)	(*n* = 32)	*p* value*
Age, years	40.7 ± 13.0 (16–65)	37.3 ± 12.8 (17–61)	0.195
Gender, female	101 (88.6)	25 (78.1)	0.128
Education, years	12.9 ± 2.8 (5–18)	12.8 ± 2.8 (6–16)	0.877
Job, yes	45 (39.5)	15 (46.9)	0.452
Household income, at least 3 million KRW/month	77 (67.5)	19 (59.4)	0.390
Married without divorce or bereavement	72 (63.2)	18 (56.3)	0.478
Age at onset, years	30.5 ± 12.3 (8–59)	29.1 ± 12.6 (11–54)	0.570
Disease duration, years	10.2 ± 8.3 (0–36)	8.3 ± 7.9 (1–33)	0.239
Attack frequency/3 months	16.0 ± 18.5 (1–90)	22.8 ± 24.9 (3–90)	0.160
Attack duration, hours	26.4 ± 21.8 (4–72)	30.1 ± 21.7 (4–72)	0.395
Migraine chronicity, chronic	64 (56.1)	20 (62.5)	0.520
Family history of migraine	70 (61.4)	21 (65.6)	0.663
Photophobia	49 (43.0)	18 (56.3)	0.183
Phonophobia	67 (58.8)	26 (81.3)	0.019
Osmophobia	55 (48.2)	17 (53.1)	0.626
GAD-7 score	3.7 ± 3.4 (0–15)	10.2 ± 5.4 (1–21)	<0.001
BAI score	9.8 ± 7.1 (0–35)	25.3 ± 14.7 (2–56)	<0.001
MIDAS, day	23.0 ± 29.3 (0–190)	36.5 ± 41.3 (0–183)	0.092
HIT-6 score	57.7 ± 7.6 (40–72)	63.9 ± 6.3 (48–78)	<0.001
MSQoL	70.1 ± 15.3 (34–94)	54.8 ± 15.3 (26–85)	<0.001

*GAD* Generalized Anxiety Disorder, *MINI-Plus 5.0.0* Mini International Neuropsychiatric Interview-Plus Version 5.0.0, *KRW* Korean Won, *GAD-7* Generalized Anxiety Disorder-7, *BAI* Beck Anxiety Inventory, *MIDAS* Migraine Disability Assessment Scale, *HIT-6* Headache Impact Test-6, *MSQoL* Migraine-Specific Quality of Life

*Independent t*-*test or chi-square tests were performed for the comparison of variables

**Fig. 1:**
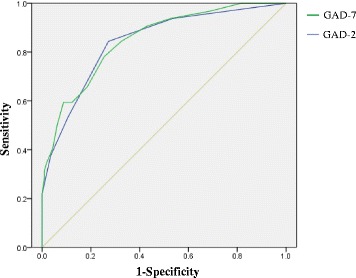
Receiver operating characteristic curves of the Generalized Anxiety Disorder-7 and Generalized Anxiety Disorder-2

The subjects completed the GAD-7 without any difficulties in comprehending and replying to the questions. Cronbach’s α for the GAD-7 and GAD-2 were 0.915 and 0.820, respectively, indicating excellent internal consistency. As shown in Table 2, all of the items in the GAD-7 were significantly and positively associated with the total GAD-7 score, and α did not decrease if items were deleted. The ROC analyses of the GAD-7 and GAD-2 are shown in Table 3, and the ROC curves are illustrated in Fig. 1. The ROC analysis of the GAD-7 exhibited an area under the curve (AUC) of 0.849 (95 % CI = 0.775–0.923; SE = 0.038; *p* < 0.001). At a cutoff score of >5, the GAD-7 sensitivity was 78.1 % and specificity was 74.6 %, with a PPV of 46.3 % and an NPV of 92.4 %. The ROC analysis of the GAD-2 exhibited an AUC of 0.842 (95 % CI = 0.763–0.920; SE = 0.040; *p* < 0.001). At a cutoff score >1, the GAD-2 sensitivity was 84.4 with a specificity of 72.8 %, a PPV of 46.6 %, and a NPV of 94.3 %.

**Table 2:** Corrected item-total correlations and Cronbach’s *α* when an item is deleted from the GAD-7

	Corrected item-total correlation	Cronbach’s α if an item deleted
Item 1	0.770	0.899
Item 2	0.824	0.893
Item 3	0.772	0.900
Item 4	0.718	0.905
Item 5	0.750	0.903
Item 6	0.722	0.904
Item 7	0.659	0.911

*GAD-7* Generalized Anxiety Disorder-7

**Table 3:** The ROC analyses of the GAD-7 and GAD-2 for the diagnosis of current GAD as determined by the MINI-Plus 5.0.0

Cut off score	Sensitivity	Specificity	PPV	NPV	AUC	SE	95 % CI	*p* value
GAD-7								
> 3	90.6	57.0	37.2	95.6	0.738	0.045	0.651–0.826	<0.001
> 4	84.4	67.5	42.2	93.9	0.760	0.046	0.669–0.850	<0.001
> 5	78.1	74.6	46.3	92.4	0.763	0.049	0.668–0.859	<0.001
> 6	65.6	81.6	50.0	89.4	0.736	0.054	0.631–0.841	<0.001
> 7	59.4	87.7	57.6	88.5	0.735	0.056	0.626–0.845	<0.001
GAD-2								
> 0	93.8	46.5	33.0	96.4	0.701	0.046	0.611–0.791	0.001
> 1	84.4	72.8	46.6	94.3	0.786	0.045	0.698–0.874	<0.001
> 2	53.1	89.5	58.6	87.2	0.713	0.058	0.600–0.826	<0.001

*ROC* reveiver operating characteristic, *GAD-7* Generalized Anxiety Disorder-7, *GAD-2* Generalized Anxiety Disorder-2, *GAD* Major Depressive Disorder, *MINI-Plus 5.0.0* Mini International Neuropsychiatric Interview-Plus Version 5.0.0, *PPV* positive predictive value, *NPV* negative predictive value, *AUC* area under the curve

The validity of the GAD-7 and GAD-2 are shown in Table 4. The GAD-7 score is well correlated with the BAI score (*p* < 0.001), the MIDAS score (*p* < 0.001), the HIT-6 score (*p* < 0.001), and the MSQoL score (*p* < 0.001). The GAD-2 score was also well correlated with the BAI score (*p* < 0.001), the MIDAS score (*p =* 0.022), the HIT-6 score (*p* < 0.001), and the MSQoL score (*p* < 0.001).

**Table 4:** Correlation between the GAD-7 and GAD-2 scores and the BAI score, the MIDAS score, the HIT-6 score, and the MSQoL score

Variable	*r*	*p* value*
GAD-7		
BAI	0.756	<0.001
MIDAS	0.231	<0.001
HIT-6	0.403	<0.001
MSQoL	−0.378	<0.001
GAD-2		
BAI	0.732	<0.001
MIDAS	0.189	0.022
HIT-6	0.336	<0.001
MSQoL	−0.310	<0.001

*GAD-7* Generalized Anxiety Disorder-7, *GAD-2* Generalized Anxiety Disorder-2, *BAI* Beck Anxiety Inventory, *MIDAS* Migraine Disability Assessment Score, *HIT-6* Headache Impact Test-6, *MSQoL* Migraine-Specific Quality of Life

*Spearman correlations are applied

## Discussion

This might be the first study investigating the reliability and validity of the GAD-7 and GAD-2 as screening instruments of anxiety in patients with migraine. The GAD-7 and GAD-2 were easily comprehended and quickly completed by patients. Furthermore, they had excellent internal consistency reliability (Cronbach’s *α* =0.915 for the GAD-7 and Cronbach’s *α* =0.820 for the GAD-2). The validity of the GAD-7 and GAD-2 was determined by correlation with scores from the BAI, the MIDAS, the HIT-6, and the MSQoL.

Many validation studies have been conducted for patients in primary care and hospital settings. The GAD-7 and GAD-2 have been validated in different populations and patient groups [15, 16, 29–31]. The initial validation study for the GAD-7, conducted in primary care patients, had a Cronbach’s *α* of 0.92, a sensitivity of 89 %, and a specificity of 82 % at a cutoff score of 9 [15]. In a Finnish study that was conducted in health centers, the sensitivity was 100 % and the specificity was 82.6 % with a cutoff score of 7 or more [29]. In a Spanish hospital-based study, the GAD-7 had a Cronbach’s *α* of 0.936, a sensitivity of 86.8 and a specificity of 93.4 % at a cutoff score of 9 [30]. In a Dutch population-based study, the GAD-7 had a Cronbach’s *α* of 0.86, a sensitivity of 83 % and a specificity of 65 % at a cutoff score of 12 or greater [31]. While the reliability in our study is consistent with these reports, the sensitivity was lower and the specificity was higher than the Dutch study at the lower cutoff score [31].

The GAD-2 has not been as frequently validated as the GAD-7. The first 2 items of the GAD-7 can be useful when an ultra-brief screening tool is desired. The initial GAD-2 validation study was conducted on primary care patients, and reported a sensitivity of 86 % and specificity of 83 % at a cutoff score of 3 or greater [16]. In a Finnish study that was conducted in health centers, the sensitivity was 83 % and the specificity was 90 % with a cutoff score of 3 or more [29]. In a Dutch population-based study, the GAD-2 had a sensitivity of 83 % and specificity of 61 % at a cutoff score of 4 or greater [31]. In our study, the sensitivity was similar and the specificity was lower than the Dutch study at the lower cutoff score [31].

The cutoff scores in the validation studies of the GAD-7 and GAD-2 in several countries were different from each other with respect to each study’s settings and language [15, 16, 29–31]. Our study showed that at a cutoff scores of 5 in the GAD-7 and 1 in the GAD-2 had the highest sum of sensitivity and specificity. Cutoff scores were all lower than in previous studies. This suggests that the GAD-7 and GAD-2 validation should be performed for each study settings and specific disease groups. For example, a validation study of the GAD-7 for patients with epilepsy in the Korea reported that a cutoff score of 6 was appropriate for detecting GAD [32]. Differences in the cutoff score may also be related to different interpretations of grading using the Likert scale according to the language difference. For example, a rapid screening instrument for detecting MDD in people with epilepsy, the Neurological Disorders Depression Inventory for Epilepsy, had different cutoff scores when it was validated in different languages [33]. Given these possibilities, it is necessary to validate the GAD-7 and GAD-2 according to different language.

There are several limitations in this study. First, the sample size of the study was small. This may caused a difference in the sensitivity and specificity compared with other studies. Second, the GAD-7 and GAD-2 consist of a self-report questionnaire. These screening instruments only provide a probable diagnosis of GAD that should be investigated by further evaluation. Third, with a cutoff score of 5 in the GAD-7 and a cutoff score of 1 in the GAD-2, the PPVs were 46.3 and 46.6 %, respectively, which may lead to false-positive results. The GAD-7 measures anxiety related problems over the past two weeks. However, the MINI interview investigates GAD over the past 6 months. Because of the difference in the observation period between the two instruments, a low PPVs of the GAD-7 and GAD-2 may exist. Fourth, the GAD-7 and GAD-2 focus on only 1 anxiety disorder, although there are many types of anxiety disorders that require clinical attention. Fifth, this study validated the Korean version of the GAD-7 and GAD-2 in Korean patients with migraine, and their diagnostic properties may be different from those in other languages and countries.

## Conclusions

Anxiety is a common psychiatric comorbidity in patients with migraine. Screening for anxiety in patients with migraine can be an effective method to recognize previously unidentified cases of anxiety. The GAD-7 and GAD-2 are simple screening instruments for detecting GAD in patients with migraine. The timely identification of anxiety in patients with migraine is important, as is proper management after diagnosis.

## Abbreviations

AUC: area under the curve
BAI: Beck Anxiety Inventory
GAD: generalized anxiety disorder
HIT: Headache Impact Test
MDD: major depressive disorder
MIDAS: Migraine Disability Assessment Scale
MINI: Mini International Neuropsychiatric Interview
MSQoL: Migraine-Specific Quality of Life
NPVs: negative predictive values
PHQ: Patient Health Questionnaire
PPVs: positive predictive values
QOL: quality of life
ROC: receiver operating characteristic

## References

[1] Stovner LJ, Hagen K, Jensen R, Katsarava Z, Lipton R, Scher A, Steiner T, Zwart JA (2007). The global burden of headache: a documentation of headache prevalence and disability worldwide. Cephalalgia.

[2] Hamelsky SW, Lipton RB, Stewart WF (2005). An assessment of the burden of migraine using the willingness to pay model. Cephalalgia.

[3] Kim SY, Park SP (2014). The role of headache chronicity among predictors contributing to quality of life in patients with migraine: a hospital-based study. J Headache Pain.

[4] Beghi E, Bussone G, D’Amico D, Cortelli P, Cevoli S, Manzoni GC, Torelli P, Tonini MC, Allais G, De Simone R, D’Onofrio F, Genco S, Moschiano F, Beghi M, Salvi S (2010). Headache, anxiety and depressive disorders: the HADAS study. J Headache Pain.

[5] Pompili M, Serafini G, Di Cosimo D, Dominici G, Innamorati M, Lester D, Forte A, Girardi N, De Filippis S, Tatarelli R, Martelletti P (2010). Psychiatric comorbidity and suicide risk in patients with chronic migraine. Neuropsychiatr Dis Treat.

[6] Sareen J, Jacobi F, Cox BJ, Belik SL, Clara I, Stein MB (2006). Disability and poor quality of life associated with comorbid anxiety disorders and physical conditions. Arch Intern Med.

[7] Lantéri-Minet M, Radat F, Chautard MH, Lucas C (2005). Anxiety and depression associated with migraine: influence on migraine subjects’ disability and quality of life, and acute migraine management. Pain.

[8] Mongini F, Ciccone G, Deregibus A, Ferrero L, Mongini T (2004). Muscle tenderness in different headache types and its relation to anxiety and depression. Pain.

[9] Hung CI, Wang SJ, Yang CH, Liu CY (2008). The impacts of migraine, anxiety disorders, and chronic depression on quality of life in psychiatric outpatients with major depressive disorder. J Psychosom Res.

[10] Merikangas KR, Angst J, Isler H (1990). Migraine and psychopathology: results of the Zurich cohort study of young adults. Arch Gen Psychiatry.

[11] Buse DC, Silberstein SD, Manack AN, Papapetropoulos S, Lipton RB (2013). Psychiatric comorbidities of episodic and chronic migraine. J Neurol.

[12] Jette N, Patten S, Williams J, Becker W, Wiebe S (2008). Comorbidity of migraine and psychiatric disorders--a national population-based study. Headache.

[13] McWilliams LA, Goodwin RD, Cox BJ (2004). Depression and anxiety associated with three pain conditions: results from a nationally representative sample. Pain.

[14] Bellini B, Arruda M, Cescut A, Saulle C, Persico A, Carotenuto M, Gatta M, Nacinovich R, Piazza FP, Termine C, Tozzi E, Lucchese F, Guidetti V (2013). Headache and comorbidity in children and adolescents. J Headache Pain.

[15] Spitzer RL, Kroenke K, Williams JB, Löwe B (2006). A brief measure for assessing generalized anxiety disorder: the GAD-7. Arch Intern Med.

[16] Kroenke K, Spitzer RL, Williams JB, Monahan PO, Löwe B (2007). Anxiety disorders in primary care: prevalence, impairment, comorbidity, and detection. Ann Intern Med.

[17] Seo JG, Park SP (2015). Validation of the Patient Health Questionnaire-9 (PHQ-9) and PHQ-2 in patients with migraine. J Headache Pain.

[18] Headache Classification Committee of the International Headache Society (2013). The International Classification of Headache Disorders. 3rd edition (beta version). Cephalalgia.

[19] Yoo SW, Kim YS, Noh JS, Oh KS, Kim CH, Namkoong K, Chae JH, Lee GC, Jeon SI, Min KJ, Oh DJ, Joo EJ, Park HJ, Choi YH, Kim SJ (2006). Validity of Korean version of the MINI-International Neuropsychiatric Interview. Anxiety Mood.

[20] Yook SP, Kim ZS (1997). A clinical study on the Korean version of Beck Anxiety Inventory: comparative study of patient and non-patient. Korean J Clin Psychol.

[21] Lee HS, Chung CS, Song HJ, Park HS (2000). The reliability and validity of the MIDAS (Migraine Disability Assessment) Questionnaire for Korean migraine sufferers. J Korean Neurol Assoc.

[22] Chu MK, Im HJ, Ju YS, Yu KH, Ma HI, Kim YJ, Kim J, Lee BC (2009). Validity and reliability assessment of Korean Headache Impact Test-6 (HIT-6). J Korean Neurol Assoc.

[23] Moon HS, Chung CS, Lee HS, Park HS, Kim SW, Woo HW (2003). The reliability and validity of the migraine-specific quality of life questionnaire for Korean migraine suffers. J Korean Neurol Assoc.

[24] Sheehan DV, Lecrubier Y, Sheehan KH, Amorim P, Janavs J, Weiller E, Hergueta T, Baker R, Dunbar GC (1998). The Mini-International Neuropsychiatric Interview (M.I.N.I.): the development and validation of a structured diagnostic psychiatric interview for DSM-IV and ICD-10. J Clin Psychiatry.

[25] Pfizer. Patient Health Questionnaire (PHQ) screeners. http://www.phqscreeners.com/. [accessed Nov 2012].

[26] Kosinski M, Bayliss MS, Bjorner JB, Ware JE, Garber WH, Batenhorst A, Cady R, Dahlöf CG, Dowson A, Tepper S (2003). A six-item short-form survey for measuring headache impact: the HIT-6. Qual Life Res.

[27] Bayliss M, Batenhorst A (2002). The HIT-6™ a user’s guide.

[28] Wagner TH, Patrick DL, Galer BS, Berzon RA (1996). A new instrument to assess the long-term quality of life effects from migraine: development and psychometric testing of the MSQOL. Headache.

[29] Kujanpää T, Ylisaukko-Oja T, Jokelainen J, Hirsikangas S, Kanste O, Kyngäs H, Timonen M (2014). Prevalence of anxiety disorders among Finnish primary care high utilizers and validation of Finnish translation of GAD-7 and GAD-2 screening tools. Scand J Prim Health Care.

[30] García-Campayo J, Zamorano E, Ruiz MA, Pardo A, Pérez-Páramo M, López-Gómez V, Freire O, Rejas J (2010). Cultural adaptation into Spanish of the Generalized Anxiety Disorder-7 (GAD-7) scale as a screening tool. Health Qual Life Outcomes.

[31] Donker T, van Straten A, Marks I, Cuijpers P (2011). Quick and easy self-rating of Generalized Anxiety Disorder: validity of the Dutch web-based GAD-7, GAD-2 and GAD-SI. Psychiatry Res.

[32] Seo JG, Cho YW, Lee SJ, Lee JJ, Kim JE, Moon HJ, Park SP (2014). Validation of the generalized anxiety disorder-7 in people with epilepsy: a MEPSY study. Epilepsy Behav.

[33] Zis P, Gatzonis S (2014). Estimating the diagnostic value of the neurological disorders depression inventory for Epilepsy in different languages. Epilepsia.

